# Current Technologies and Recent Developments for Screening of HPV-Associated Cervical and Oropharyngeal Cancers

**DOI:** 10.3390/cancers8090085

**Published:** 2016-09-09

**Authors:** Sunny S. Shah, Satyajyoti Senapati, Flora Klacsmann, Daniel L. Miller, Jeff J. Johnson, Hsueh-Chia Chang, M. Sharon Stack

**Affiliations:** 1 Department of Chemical and Biomolecular Engineering, University of Notre Dame, Notre Dame, IN 46556, USA; sshah1@nd.edu (S.S.S.); ssenapat@nd.edu (S.S.); 2 Bionics Innovation Center Nonprofit Ltd., 1088 Budapest, Hungary; flora.klacsmann@gmail.com; 3 Department of Pathology, The Johns Hopkins University School of Medicine, Baltimore, MD 21205, USA; miller.climb@gmail.com; 4 Harper Cancer Research Institute, University of Notre Dame, South Bend, IN 46617, USA; jjohns39@nd.edu; 5 Department of Chemistry and Biochemistry, University of Notre Dame, Notre Dame, IN 46556, USA

**Keywords:** human papillomavirus, cervical and oropharyngeal cancers, diagnostics, integrated platform, HPV DNA, *E6/E7* genotypes, microRNA

## Abstract

Mucosal infection by the human papillomavirus (HPV) is responsible for a growing number of malignancies, predominantly represented by cervical cancer and oropharyngeal squamous cell carcinoma. Because of the prevalence of the virus, persistence of infection, and long latency period, novel and low-cost methods are needed for effective population level screening and monitoring. We review established methods for screening of cervical and oral cancer as well as commercially-available techniques for detection of HPV DNA. We then describe the ongoing development of microfluidic nucleic acid-based biosensors to evaluate circulating host microRNAs that are produced in response to an oncogenic HPV infection. The goal is to develop an ideal screening platform that is low-cost, portable, and easy to use, with appropriate signal stability, sensitivity and specificity. Advances in technologies for sample lysis, pre-treatment and concentration, and multiplexed nucleic acid detection are provided. Continued development of these devices provides opportunities for cancer screening in low resource settings, for point-of-care diagnostics and self-screening, and for monitoring response to vaccination or surgical treatment.

## 1. Global Burden of Cervical and Oropharyngeal Cancers

Globally, cervical cancer is the third leading cause of cancer death among women, with >550,000 new cases diagnosed annually, resulting in >288,000 deaths per year [1]. Cervical cancer affects relatively young women from 30 to 50 years of age who are often engaged in family care and/or are employed outside the home, resulting in a greater years-of-life loss relative to other female cancers with later onset. In developing nations, the mortality from cervical cancer is up to 50-fold higher than for U.S. women [2,3]. U.S. cervical cancer incidence and death rates have declined by >60% [4,5] due to access to screening to identify early stage precancerous lesions, treatment of precancerous lesions prior to progression and, more recently, due to vaccination [6]. Although with early detection up to ≈90% of cervical cancers are curable [7,8], developing nations lack access to regular health exams and a formal healthcare system, significantly compromising early detection of cervical cancer. The primary etiologic agent of cervical cancer is the human papillomavirus (HPV). HPV genotypes that infect the human genital tract are classified as “high” or “low” risk based on potential carcinogenicity [1]. Persistent cervical infection with oncogenic high risk HPV, particularly HPV16 and 18, is the cause of the vast majority of cervical cancers [9,10,11]. However, geographic differences in prevalence of oncogenic high risk HPV types are widely reported and type-specific prevalence can change post-vaccination, highlighting the need for screening tools that detect specific HPV-related molecular signatures [5,12,13,14,15].

In addition to the genital tract, HPV infects other anatomic sites, including the oropharynx (tonsillar area). U.S. incidence of HPV-associated oropharyngeal squamous cell carcinoma (OPSCC) is predicted to surpass that of cervical cancer by 2020 [16]. Overall, head and neck squamous cell carcinoma (HNSCC), which includes tumors of the oral cavity, larynx and oropharynx, is the 6th most prevalent cancer worldwide, representing a major global health problem, with >400,000 cases and >200,000 deaths annually [17]. Owing to public health efforts encouraging smoking cessation, the overall incidence of HNSCC has decreased in the U.S. by 50% since 1984 [16,18]. In striking contrast, during the same time period the incidence of OPSCC has increased by 225%, predominantly in a significantly younger patient demographic [16]. Molecular analyses of OPSCC have shown that 40%–80% of OPSCC diagnosed in the U.S. and 18% of OPSCC worldwide contain HPV [19,20], and numerous studies support a viral etiology for oral cancer in these sites [21,22,23,24,25]. Most HPV positive oral tumors arise deep within the crypts of the tonsils, are obscured from gross visualization, and often present with lymph node metastases [26], further supporting the need for new screening modalities that can detect HPV-associated disease in biological samples.

## 2. Human Papillomaviruses

HPVs are small, 50–55 nm in diameter, non-enveloped double-stranded DNA viruses that infect the skin and mucous membranes, causing both benign and malignant conditions. Worldwide, ≈5% of the total global cancer burden is attributable to HPV [3]. Many HPV types are spread by sexual contact (vaginal, anal or oral sex), while others are not sexually transmitted. The sexually transmitted HPVs are generally classified as “low risk” or “high risk” based on potential carcinogenicity and are the most common sexually transmitted viruses in the U.S. [1]. Low risk HPVs cause genital warts and papillomatosis in the respiratory tract. In contrast, high risk HPVs can be oncogenic, leading to HPV-associated cancer. The majority of high risk HPV infections are cleared by the immune system within 1–2 years and are asymptomatic. However, persistent infection with high risk HPV can induce cellular changes leading to cancer. The most common HPV-associated cancers are localized in the cervix and oropharynx, although anal, penile, vaginal, and vulvar cancers are also linked to HPV [4]. It has been postulated that the common endodermal origin of the squamous lining of both the cervix and tonsils, their shared locations at the junction between external and internal environments, and the human microbiota at these mucosal surfaces play a role in enabling persistent HPV infection, providing a favorable milieu for carcinogenesis [27].

### 2.1. Genes Encoded by HPVs

**Figure 1:**
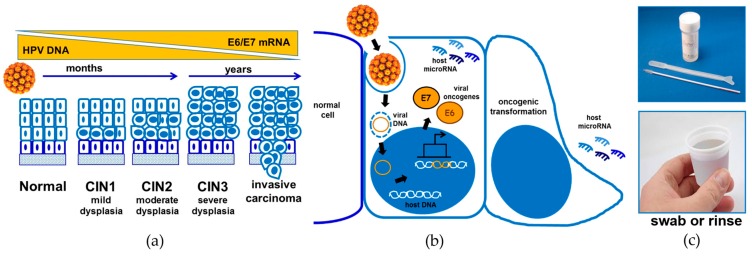
(**a**) Timeline of cervical cancer progression. Human papillomavirus (HPV) infects the basal cells of the cervical epithelium (dark blue) exposed by wounding, leading to cytological abnormalities; (**b**) Expression of viral oncogenes *E6/E7* accompanies malignant transformation; (**b**) HPV infection pathway. Following viral entry, viral replication is synchronous with host cellular DNA replication. Integrated and episomal viral DNA produce E6 and E7 oncoproteins. E6 targets p53 for degradation and prevents apoptosis while E7 inactivates retinoblastoma (Rb) tumor suppressors, promoting cell cycle progression. The end result of these processes is cellular transformation. HPV infection also alters transcription of host microRNAs; (**c**) Samples for detection of HPV E6/E7 mRNA or host microRNA. CIN: cervical intraepithelial neoplasia.

HPVs encode only eight genes, which enable host infection and viral replication. HPV infection initiates in proliferating basally-localized mucosal keratinocytes, often at sites of injury (Figure 1). The genome contains six early genes (*E1*–*E7*) that support viral DNA replication and two late genes (*L1* and *L2*) that encode capsid proteins necessary for viral assembly and release. The compact viral genome necessitates viral dependence on host factors to carry out the life cycle, thus viral replication is linked to that of host cells [27,28]. Virally encoded oncogenes do not cause direct transformation of host cells, but induce immune evasion and aberrant cellular proliferation scenarios that support genomic structural aberrations and mutations [29]. In particular, the viral oncogenes *E6* and *E7* cooperate to induce cellular immortalization and genomic instability, affecting multiple factors involved in cell cycle progression, cell survival, differentiation, and inhibition of apoptosis [30]. Corrupted DNA damage response and repair pathways also cooperate to promote a cellular environment tolerant to mutagenesis and ultimately tumorigenesis [28,29].

In the cervical epithelium, a productive infection initiates with the onset of viral replication leading to low-grade cervical intraepithelial neoplasia (CIN) characterized by cytological abnormalities (CIN1, CIN2 with mild to moderate dysplasia, respectively). Persistent HPV infection results in progression to high-grade lesions (CIN3, invasive carcinoma). In productively infected cells, transcription of viral oncogenes *E6* and *E7* in host cells mediates degradation of the tumor suppressors p53 and retinoblastoma (Rb) and alters the activity of proteins that regulate cell cycle, leading to cellular transformation (Figure 1). The majority of HPV infections are asymptomatic and resolve within two years. It is currently unclear why some infections resolve while others result in neoplasia, but it may be related to host factors. In women with normal immune systems, cervical cancer development occurs over a timeframe of 15–20 years. Development is accelerated in women with weakened immune systems, such as those with untreated human immunodeficiency virus (HIV) infection [31].

### 2.2. Role of MicroRNAs in HPV-Associated Cancer

In addition to research on HPV-encoded oncogenes such as *E6* and *E7*, the molecular-level response of the ‘host’ to an oncogenic HPV infection is an area of active investigation. MicroRNAs (miRs) can function both as oncogenes and tumor suppressors. Both upregulation of oncogenic miRs and loss of tumor suppressive miRs in host cells have been reported in HPV-associated malignancies [26,32]. Expression profiling of cancers and functional studies performed in cancer cell lines and pre-clinical models have revealed provocative patterns implicating miR dysregulation in oncogenesis and tumor progression. As a single miR has the ability to regulate hundreds to thousands of genes, induction or loss of miR expression is predicted to dramatically alter cell behavior. In addition, evidence suggests that miRs are dysregulated at early stages of cancer initiation and progression [33], suggesting that longitudinal monitoring of specific host miRs that are altered in the presence of an active oncogenic HPV infection may provide a novel mechanism by which to distinguish latent from active infections.

Multiple miRs have been reported as dysregulated in HPV-associated cervical and oropharyngeal cancer, summarized in recent reviews [27,34]. Mechanisms for dysregulated miR expression include chromosomal alterations that delete or amplify a miR locus, epigenetic regulatory factors, or altered transcription factor regulation [35,36,37]. Using multiple cohorts of human OPSCC tumors, a panel of miRs (designated oncogenic miR panel, comprised of miR-9, miR-106b and miR-93) that differentiate HPV+ OPSCC from HPV-disease has been identified [26,27]. This panel thus represents the host response to an oncogenic HPV infection, as the virus does not produce its own miRs. It is interesting to note that the miR-17~92 cluster and its genomic paralogue miR-106b~25 are upregulated in both cervical and HPV-associated OPSCC (HPV+ OPSCC) [33,38]. Strong upregulation of miR-9 is also observed in HPV+ OPSCC but not in OPSCC that are HPV-negative [27]. The specific mRNA targets of these miRs and the functional consequences of altered target gene expression are currently under active investigation. However, these specific miRs, upregulated in host cells by an active oncogenic HPV infection, represent a target for improved early detection of HPV-associated malignancies.

It should be noted that a given miR is not specific for a single malignancy, highlighting the need for multiplexed analysis of a panel of miRs to potentially enhance the specificity of disease detection. Although the majority of miRs are intracellular, some miRs are also present in the circulation where they may circulate bound to specific proteins or as components of exosomes [39,40]. Detection of miRs, whether in a cell lysate or a biofluid, is facilitated by the fact that miRs are highly stable to extremes of temperature, pH and RNases, likely due to binding to carrier proteins or lipids [41].

### 2.3. The Need for Cancer Screening

HPV+ OPSCC are associated with distinct survival strata, thus early identification of this patient cohort is needed to tailor care to the unique biology of HPV+ tumors [18]. Current screening approaches do not distinguish between the simple presence of the non-integrated HPV vs. a cancer-causing oncogenic HPV infection. It is estimated that there are 14 million new genital HPV infections in the U.S. each year [42], with 80%–90% of sexually active men and women infected at some point during their lifetime, 40%–45% of which are infected with high risk HPV [43,44]. Similarly, 26 million Americans are estimated to have an active oral HPV infection, with 6.2 million new infections yearly. Of the millions of people with an HPV infection, most will not develop cancer, but given the millions of young men and women harboring oral HPV and the positive clinical outcomes associated with early detection, development and validation of a technically and conceptually novel multi-target nucleic acid sensor platform for early detection of HPV-associated OPSCC is warranted.

Due to the long clinical latency between infection and oncogenesis (often tens of years) and the lack of clinical treatment for persistent HPV infections that have not yet induced abnormal cellular changes, the availability of technologies for monitoring of viral oncogene expression (*E6,E7*) or host miR response to an oncogenic HPV infection (miR-106b, miR-9) in biological fluids, such as a cervical swab or oral rinse, would facilitate early discovery of malignant transformation and thus improve patient outcomes. In the case of cervical cancer, it is relevant to note that the vast majority (>85%) of cervical cancer deaths are in women in developing countries that also lack access to routine cervical cancer screening or vaccination against HPV infection [45]. Although sophisticated diagnostic instruments located in centralized laboratories can provide highly sensitive, selective and reproducible results, many of these settings in developing nations lack the necessary infrastructure. If available, such infrastructure is only accessible at hospitals in major cities. The shipping of samples to central clinical laboratories is time consuming, expensive and prolongs diagnosis and proper therapy. Thus, there is a significant unmet need for rapid, low cost, nucleic acid-based diagnostics capable of detecting E6 and E7 mRNA or host miRs such as miR-106b or miR-9. An ideal cancer screening platform must have the following characteristics that make it amenable for developed and developing countries:

1. Low cost: A sensor that is developed using simple chip fabrication steps can be mass-produced, thus making it amenable for low-resource settings.
2. Portability: The platform should be portable and easy to operate with the possibility for battery operation and minimal need of infrastructure.
3. Simplicity of assay protocol: An easy-to-use device that is portable will eliminate the need for a sophisticated clinical laboratory facility or highly skilled, trained personnel. The final integrated platform needs to be an automated sample-to-answer device.
4. Signal stability: A sensing signal that is stable, reproducible and insensitive to surrounding noise.
5. Sensitivity and selectivity: A sensor should be sensitive enough for the target of interest and have the requisite specificity comparable to the gold standard.
6. Rapid and robust: The device should generate results rapidly, allowing medical personnel to diagnose disease and start immediate treatment (screen-and-treat). Additionally, the platform should be robust and not require any refrigeration or other external equipment to perform the test.
7. Multiplexing: The platform should be flexible enough to detect more than one target biomarker.

## 3. Established Tools for Screening Cervical and Oropharyngeal Cancers

Established techniques include conventional cytology or histology, liquid-based monolayer cytology, HPV testing, visual inspection of the cervix (with or without acetic acid, or VIA) or the oral cavity, light-based detection systems for oral cancer screening, or HPV-associated serologic markers (HPV biomarkers). These have served extensively for screening of cervical and oropharyngeal cancers and remain in use today. Table 1 summarizes these techniques and they are reviewed in greater detail in the following sub-sections.

### 3.1. Conventional Cytology (Pap Test)

The Pap test (Pap smear, cervical smear) is the oldest known test that has served as the standard for cervical cancer screening. Since its introduction into standard clinical use in the 1950s, this method has reduced death from cervical cancer by 60% in women under 55 [51]. The Pap smear is performed by scraping epithelial cells from the surface of the cervix followed by an examination under a microscope to observe abnormalities. Patients with a positive screening test are recommended to undergo a secondary evaluation using colposcopy, with results reported as normal, probable low-grade or high-grade precancerous lesions, or invasive cancer [52]. Disadvantages associated with conventional cytology methods include the extremely laborious process, long assay-to-answer timeline, the necessity for experienced and trained personnel as well as the need for continuous rescreening and quality control. The cytology method may have high specificity (≈80%), however, the sensitivity of the method for detecting cervical intraepithelial neoplasia is just above 50% [53,54]. The method inevitably detects abnormalities that will never progress to life-threatening disease which means that over 80% of women with high grade cervical intraepithelial neoplasia will not develop invasive cancer [55]. In the U.S., a Pap test itself costs $20–$30, but the costs for Pap test visits can range up to $1,000, largely because additional tests that may or may not be obligatory are often added [56]. Recognizing the nature of the problems associated with screening, an integrated, computer-assisted imaging system was approved by the Food and Drug Administration (FDA) for automated screening of Pap smear cytology samples. In a study prior to FDA approval, the ThinPrep^®^ Imaging System (TIS) (Cytyc Corporation, Marlborough, MA, USA) showed a significant improvement in specificity for detection of high grade lesions, however, concerns remain regarding the overall cost of assay per test and its utility in low-resource settings [57]. Sensitivity of the ThinPrep^®^ system for detecting high grade lesions was ≈76% compared to ≈68% detection sensitivity when conventional Pap smear test was utilized. Additionally, ThinPrep had greater specificity (86%) compared to the conventional method (79%) [58].

### 3.2. Liquid-Based Monolayer Cytology

Monolayer cytology has been in use since the 1990s. The main difference from the conventional Pap method is the liquid storage of the cervical specimen in a solution that preserves cellular morphology while lysing other components in the sample including blood cells and microbes. Many cross-sectional studies have been performed related to conventional and monolayer cervical smear [59,60]. The liquid sample has the advantage of being suitable for high-risk HPV testing and may reduce the occurrence of unsatisfactory specimens [60]. However, interpretation of the results for liquid-based cytology differs from the conventional Pap test, thus requiring additional training of cytologists and increased costs. Despite the higher cost per test, the liquid-based method has replaced the older (and cheaper) slide test in many countries [56]. Several monolayer-based systems are on the market (ThinPrep^®^, MonoPrep^®^ and SurePath^®^) and studies have shown that the sensitivity is improved by 1.29× for the monolayer technology compared to the conventional cytology method for high grade squamous disease [61,62].

### 3.3. Visual Inspection with Acetic Acid (VIA)

VIA is predominantly used in low-resource settings. It is a quick and simple visual inspection of the cervix using acetic acid (white vinegar; VIA or Lugol’s iodine; VILI) to highlight precancerous lesions. The procedure eliminates the need for laboratories and transport of specimens, requires very little equipment and provides women with immediate test results (one minute after the application of 4% acetic acid) [52]. As a screening test, VIA may perform as well as cervical cytology in accurately identifying pre-cancerous lesions, but cytology provides fewer false positives relative to VIA [63]. This has been demonstrated in various studies where trained physicians and mid-level providers correctly identified women at high risk of developing cervical cancer with VIA and VILI, with sensitivity ranging from 67% to 79% for VIA and from 78% to 98% for VILI [64]. Similarly, the specificity among several different studies ranged from 64% to 86% and 73% to 91% for VIA and VILI, respectively [64]. This great disparity in sensitivity indicates one of the main limitations of visual inspection methods as results generated by VIA are highly dependent on the accuracy of an individual’s interpretation. This mostly results in overdiagnosis and overtreatment and a low net predictive value [65]. Factors that may affect proper diagnosis include inflammation, cervical condyloma and leukoplakia, which can result in false positive results [66]. Another limitation of visual tests is that they are not reliable in postmenopausal women due to changes in the endocervical junction, the area in which precursors of cervical cancer arise [63]. Nevertheless, VIA is a promising approach, but initial training and ongoing quality control are of paramount importance to be considered for public health initiatives. Despite the deficiencies, it should be noted that VIA is predominantly used in developing nations for performing large-scale cervical cancer screening due to its cost-effectiveness [67].

### 3.4. Serological Testing of HPV

Current efforts aimed at appropriate targeting for immune therapy for cervical cancer are associated with HPV. Serologic markers could be tumor-specific antigens associated with expression of the *E6* and *E7* oncogenes associated with high-risk HPV [68]. While circulating cervical cancer antibodies have been identified, the value of *E6* and *E7*-specific serology for the diagnosis of this disease is still questionable [68]. Another study showed genital HPV infection frequently induces antibody-mediated immune responses, mostly directed against the viral capsid [48]. Immunological assays based on the use of HPV capsids are known to detect type-restricted antibodies, possibly due to the presentation of type-specific conformational epitopes. Thus, specific antibodies have been found to react against peptide sequences on viral capsid proteins of both high- and low-risk HPV types [48]. Also immunization with heat-shock proteins (HSPs) derived from virus infected tumor cells has been shown to induce potent antitumor or antiviral immunity [68]. Since it is still unclear whether the type of HPV infection, the activity of the infection, or the biological response to the infection could be clinically useful for predicting prognosis of cervical carcinoma patients, the practical adaptability of this method remains preliminary [69].

### 3.5. Oral Cancer Diagnostic Technologies

Routine oral cancer screening is important and has been shown to significantly reduce mortality [70]. Screening for oral cancer is commonly performed at dental clinics or physician’s office by a routine visual inspection of the oral cavity using a normal flashlight or white light illumination, followed by palpation of suspicious lesions. The trained clinician can often identify subtle changes in early lesions, but general dentists or physicians may lack the proper knowledge, resulting in false diagnoses [71]. Several visualization adjuncts to standard oral examination are now commercially available such as toluidine blue or ViziLite [72,73]. ViziLite uses a chemiluminescence light to detect abnormal surface changes in the oral cavity [74]. ViziLite employs a 1% acetic acid rinse to make cell nuclei in the epithelium more prominent thus leading to the appearance of abnormal epithelium as exceedingly white under chemiluminescence light. While ViziLite has the potential to detect malignant disorders, it has poor sensitivity (≈77%) and specificity (≈28%) for detecting dysplastic lesions [75]. Other light-based detection systems use blue/white LED and autofluorescence as light sources. They are designed to detect possible abnormalities in the epithelial tissue that are not necessarily visible to the naked eye. Technologies like wide-field fluorescence imaging and high resolution optical techniques are emerging to improve the visualization of neoplastic regions. Wide-field imaging (like VELscope from LED Dental, White Rock, BC, Canada) enables rapid inspection of large mucosal surfaces, to aid in the recognition of suspicious lesions. Neoplasia is associated with a loss of autofluorescence and can reveal lesions that are difficult to detect with standard white light examination. However, several reports suggest that the presence of inflammation may be associated with loss of stromal autofluorescence captured by the wide-field imaging technique, generating false positive results [71]. Additionally, a study has shown the deficiency of VELscope in its ability to distinguish between high-risk and low-risk lesions with sensitivity and specificity for the detection of a dysplastic lesion to be ≈84% and ≈15%, respectively [76]. On the contrary, high-resolution optical imaging techniques allow identification of epithelial changes at the subcellular level, resulting in the ability to distinguish cancer-related malignant cell lesions from conventional inflammatory conditions [71]. Another advantage of this technique includes its ability to image in vivo oral tissues with subcellular resolution, making the diagnosis more reliable [71]. Carlson et al. reported a dual-mode reflectance and fluorescence confocal microscope (DCM) to record molecular images of tissue as well as tissue architecture and cellular morphology [77]. Images obtained with the combination of reflectance and fluorescence provide information about both the morphologic and molecular changes associated with cancer progression. Other marketed visual inspection devices for early screening include the BrushTest (OralCDx, Suffern, NY, USA) and Microlux (adDent, Danbury, CT, USA); however, they exhibit low specificity, high false positive/false negative rates and may not be well suited for detection of cancers in the oropharynx [78,79]. A study concluded that the OralCDx test demonstrated sensitivity of ≈73% and specificity of ≈92% with a positive predictive value of ≈89% and a negative predictive value of ≈80%. Despite the promising results, the authors concluded that the most reliable method of screening of lesions is the conventional biopsy technique [80]. While the gold standard for oral cancer diagnosis is biopsy followed by assessment of the tissue by a pathologist, this procedure is not easily performed in the dental clinic or in low-resource settings [81,82]. Thus physical visualization of the oral cavity may result in early detection, however, a high percentage of oral cancers remain undiagnosed until advanced stage, highlighting the need for better early cancer screening tools [70,83].

## 4. Nucleic Acid-Based Screening Tools

While the traditional tools for cervical and oral cancer screening continue to be widely used, recent developments for several lab-based non-invasive methods have been made to uncover differential expression levels of miRs that may more accurately report on physiological status or disease progression. Some of the commonly available techniques include (i) microarray; (ii) quantitative reverse transcription-polymerase chain reaction (qRT-PCR) and (iii) next-generation sequencing. Table 2 summarizes the performance specifications of these techniques in relation to the criteria described above.

### 4.1. Microarray-Based Sensing

Microarray is one of the most powerful high-throughput techniques capable of monitoring the expression of thousands of miRs in a single experiment [85,86,87,88,89,90]. The experimental process includes construction of an array of thousands of probes on a microarray slide by photolithography, allowing parallel tracking of all targets in a sample. The end modified fluorescently-labeled miRs are hybridized with the probes printed on the array and the brightness of the spot is then accounted as a captured target signal. Differentiation between closely-related miR sequences can be problematic with other techniques such as qRT-PCR (discussed below), however, careful selection of control probes, stringent washing and hybridization conditions and proper data analysis provide advantages in terms of specificity [91,92]. Specificity can be further improved by incorporating locked nucleic acid (LNA) probes that increase the thermal stability of duplexes and normalize melting temperatures across all of the captured probe–target duplexes [93,94,95]. Despite having the advantages of low cost per test per target and screening of multiple targets simultaneously, the microarray technology lacks sensitivity, dynamic range and ability for absolute quantification when compared to other nucleic acid screening technologies including qRT-PCR or RNA sequencing. Additionally, detection of miRs with low copy number requires long assay time (due to diffusion limitation) to allow the target to bind to the probe printed on the microarray slide. This long assay time enhances the false positive signal, as non-target molecules with large copy numbers have higher probability to non-specifically bind to the probes [96]. Low reproducibility of the assay is an additional limitation, thus requiring a study with a large number of replicates [97]. Some of the commercially-available microarrays include the Agilent oligonucleotides microarray, Affymetrix GeneChip miRNA array and Exiqon miRCURY LAN miRNA array [98,99,100]. However, several studies have reported poor correlation between these commercially-available technologies and other microarray and qRT-PCR techniques [101].

### 4.2. Polymerase Chain Reaction-Based Sensing of HPV DNA

Although qRT-PCR requires extreme care to avoid human sample handling error and contamination, it can be considered as a gold standard due to its superior performance in terms of sensitivity, specificity and quantification ability relative to other available miR profiling methods. In qRT-PCR, the small miR is reverse transcribed to cDNA, followed by conventional qPCR. The amplification is achieved by using a miR-specific probe and a stem-loop/poly(A) primer. Either the SYBR Green or TaqMan technique is employed to measure the fluorescence signal of the amplicon. This technique is moderately expensive, relatively sensitive, specific and capable to quantify compared to microarray [102]. Two of the limitations of this method are the difficulty in specific probe design due to the short length of miRs and differentiation of miR sequences by a handful of bases. Closely-matched sequences make the melting temperatures very similar, resulting in a higher probability for non-specific and differential amplification efficiency of the designed primer [103,104]. Furthermore, lack of high-throughput capability is another limitation for large miR studies.

PCR-based detection of miR is technically challenging and costly and has not been used to screen for OPSCC. Salivary diagnostics based on PCR DNA detection (such as OraRisk HPV from OralDNA Labs) are available to detect the presence of the virus [105]. A disadvantage of this approach is that it cannot discriminate between transient infections (with normal cytology) and high grade lesions that need treatment, potentially leading to overdiagnosis of lesions that would have spontaneously regressed [106]. A variety of new protocols have been reported to detect additional nucleic acid biomarkers associated with high-risk HPV. Detection of mRNA encoding the E6/E7 oncoproteins preserves the analytic sensitivity yet correlates with lesion severity (Figure 1a) and is thereby more appropriate for risk evaluation [107,108]. There are several tests commercially available in the market for detection of HPV DNA or the *E6/E7* oncogenes. Qiagen’s Digene Hybrid Capture^®^ 2 HPV DNA test (Valencia, CA, USA) [109], Hologic Cervista^®^ HPV HR (Marlborough, MA, USA) [110], Cervista^®^ HPV 16/18, Cobas^®^ HPV Test by Roche (Indianapolis, IN, USA) [111] and Aptima^®^ by GenProbe (Woburn, MA, USA) [112] are the five FDA-approved HPV assays and are compared below in Table 3. However, several other HPV tests are also available in the market, e.g., Amplicor^®^ HPV Test by Roche, Clart human papillomavirus 2 by Genomica (Madrid, Spain) [113], INNO-LiPA by Innogenetics (Ghent, Belgium) [114], Genomica’s Clinical arrays^®^ HPV [115] and Abbott’s RealTime High Risk HPV assay (Chicago, IL, USA) [116]. One other commercially available platform to detect mRNA for the *E6/E7* oncogenes is PreTect^®^ Proofer by Norchip (Klokkarstua, Norway) [116]. However, all these detection methods require access to highly specialized and bulky instrumentation for sample pretreatment and detection purposes. For example, the Cobas^®^ HPV system weighs over 150 kg and is 166 cm wide while the Hologic Panther system weighs an incredible ≈363 kg and is over 175 cm in height [117,118]. The bulkiness of such systems is not a concern in developed nations with strong healthcare infrastructure but may not be suitable for developing nations with poor infrastructure where cases of HPV-associated cancer may be more prevalent. Both traditional methods like VIA and PCR-based methods for HPV detection can be used in a “screen-and-treat” approach, wherein positive results initiate cryotherapy treatment [17], a relatively low-technology treatment with high efficacy and minimal morbidity. This screen-and-treat approach was shown to be safe and effective with a high risk-benefit ratio [119].

### 4.3. Next-Generation Sequencing

Despite the expense, high-throughput next-generation sequencing is becoming the dominant technology in the discovery and experimental validation of miR biomarkers, as it can distinguish between closely related miR sequences. The process starts with the preparation of a cDNA library from the RNA sample, followed by the sequencing of millions of individual cDNA molecules from the library. The sequence reads are then aligned against a miR sequence database after extensive bioinformatics processing to identify the known and unknown miRs present in the sample. The number of sequence reads thus make the technique very quantitative. This technique is suitable for both new miR discovery and study of known miRs. However, one main disadvantage of next-generation sequencing is that it shows the same biases in quantification analysis due to involvement of enzyme processing of the miRs, similar to qRT-PCR and microarrays, which can result in misrepresentation of the miR complement [104]. Some of the commercially available platforms include the Roche (454) Genome Sequencer that can simultaneously read 1 million sequences in excess of 400 bp, the Illumina (Solexa) Genome Analyzer (San Diego, CA, USA) that is capable to analyze 200 million sequences of up to 100 bp and the Applied Biosystems SOLiD (Foster City, CA, USA) system that can perform 400 million sequences read of 50 bp [129]. However, sequencing remains too expensive for routine use, even with the emerging protein-nanopore technologies [130].

### 4.4. Challenges with Current Nucleic Acid Screening Tools

Many challenges still remain to be addressed if the recent discoveries in miR biomarker research are to be successfully advanced, particularly related to the detection accuracy and reproducibility of the quantified results. Although it is better to profile miR expression by microarray followed by qRT-PCR, it is difficult to validate and correlate miR expression data obtained using different profiling methods [131,132]. Higher variation in reproducibility was also observed in both assay platforms for low miR copy number samples. Mestdagh et al. reported a relative higher RT variation with and without pre-amplification for low copy number (*R*^2^ = 0.685) compared to moderate (*R*^2^ = 0.806) and highly (*R*^2^ = 0.806) expressed miRs [133]. Further, higher false positive results were detected with the microarray compared to the qPCR-array [132]. A comparison of qPCR and microarray assays as reported by Chen et al. during their study for miR expression profiling demonstrated good reproducibility (*r* = 0.974) of microarray platform for duplicate miR samples, but observed considerable variation (*r* = 0.443) in the results associated with two assay platforms when analyzing 84 miR samples [132]. Similarly, poor correlation (44%) between microarray and qRT-PCR miR expression results were reported in 49 samples from lung cancer cases [84]. Several other studies also show relatively poor correlation [134,135,136]. The higher dynamic range and higher specificity of qRT-PCR compared to microarray suggests the potential for poor correlations in the non-overlapping specificity and dynamic regions. Additional error may be introduced during sample handling and interfering agents or inhibitors that vary between the two techniques. The techniques also have completely different assay protocols [137]. The hybridization equilibrium dissociation constant K_D_ and the PCR efficiency are highly temperature-sensitive and length-dependent. Hence, as the two techniques involve different reacting species and kinetic mechanisms at different reaction times and temperatures, variability in the hybridized and amplified yield is not surprising. While alternative detection strategies that address these issues are needed, screening via Pap smear, VIA and qPCR are the current options for large-scale screening in the developed and developing world.

### 4.5. Advancements in RNA and Electrochemical Sensing

The molecular diagnostic research community has been actively engaged for years in the development of a highly sensitive biosensing platform for cancer biomarker detection. These biosensing assay platforms use a probe-functionalized sensing element where the target capturing event is measured most commonly by means of an optical or electrical signal. Among the reported optical based quantification technologies, most rely on fluorescence labeling such as a molecular beacon [138], Förster resonance energy transfer [139,140] and dye-trapping liposomes [141]. In a laboratory with sophisticated optical facilities, these techniques can quantify down to single-molecule resolution. Quite often, however, such precise quantification is only possible for captured or confined targets isolated from the sample and quantification of the targets in the original sample must still be estimated by extrapolation and correlation, as the capturing and confinement rate is kinetic- or transport-limited. While there is abundant literature on DNA sensing technologies, little is reported about quantification of miR expression, particularly using technologies amenable to a low-resource setting. A group of researchers have developed a power-free microfluidic chip capable of optically amplifying and detecting miR targets with a detection limit of 0.5 pM from a 0.5 µL sample solution in under 20 min [142]. Another group has utilized the concept of fluorescence quenching using gold nanoparticles to detect label-free miR molecules with a detection limit of 3.8 pM and a dynamic range up to 10 nM [143]. A recent study used the phenomenon of conformational change of stem-looped aptamers to specifically capture target miR and amplify the detection signal by 50-fold [144]. The current standards for miR profiling like RT-PCR and microarray technologies are lab-bound and suffer from normalization issues that do not allow for cross-reference [145]. Although portable PCR units are now available, the extensive pretreatment for miR reverse-transcription has become a major bottleneck for low-resource applications. While practical portable optical detectors are available, however, their sensitivity for miR quantification is often too inadequate to justify their higher cost. Amplification of the optical signals with plasmonic and scattering nanostructures are widely reported but they remain too costly for large scale miR biomarker study and also suffer from imprecise correlation between the sample target number and the number of targets captured by the nanostructures [146]. Thus, all these techniques are labor-intensive, expensive and not realistic for miR detection in biological samples in low-resource settings.

Capacitance, conductance and field-effect transistor (FET) electrode sensors are typically sensitive to sample ionic strength, as the thickness of the screening electrical Debye layer is comparable in dimension to the target nucleic acid molecules [147,148]. The largest drawback of all sensing platforms is their long assay time due to diffusion-based transport of large nucleic acid molecules to the surface [149] and the corresponding normalization issue with the true target number in the sample, making them unsuitable for miR profiling. Several techniques have been suggested to remove the slow transport of large nucleic acid molecules to the sensor. One involves the activation of high voltage at the electrode sensor to electrophoretically attract nearby DNAs [150]. However, this electrophoretic concentration technique is non-specific and the elevated voltage can produce undesirable faradaic reactions for high-ionic strength buffers resulting in false current or voltage signals. Nanoslot sensors with short transport time have also been reported but they are too expensive and labor intensive and hence not practical for profiling of large libraries of miRs [151]. Further, all the existing nucleic acid detection technologies require purified sample free of interfering agents and controlled pH/ionic strength thus rendering them ineffective for field applications or miR profiling. Thus a simple and efficient integrated platform capable of isolation and detection of nucleic acid biomarkers remains a significant unmet need in medical diagnostics.

## 5. Recent Developments in Integrated Lab-on-a-Chip Technologies for Nucleic Acid Screening

While there are several nucleic acid-based screening platforms available for detection of HPV DNA, *E6/E7* genes or miR biomarkers, there is a significant need for integrated systems that provide rapid results in a sample-to-answer format. Microfluidic systems currently under development afford the advantage of forming such an integrated system due to flexibility in design and the necessity for small volumes. Additional advantages are realized in low-resource settings including portability, possibility of automation to reduce the need for personnel, decrease in power consumption and easy disposability. Modular multi-stage microfluidic platforms with sensors can also offer standardized pretreatment units for different samples thereby eliminating the need for steps like centrifugation that are not suitable for field applications and even PCR amplification which introduces normalization issues. While offering these advantages, microfluidic systems do not compromise on the sensitivity and selectivity of the assay [152]. However, overly complex integrated units with multiple connected microchannels are not practical as they introduce contamination and clogging. Hence, a simple integrated unit with high robustness will likely be the first commercial nucleic acid sensing platform for point-of-care applications. The two main components of an integrated platform include a sample processing unit capable of extracting nucleic acid biomarkers from a target sample coupled with a biosensing unit capable of sensing the extracted biomarkers.

Normalization and variability issues can be minimized with sample selection and improved/standardized sample processing techniques and, if possible, elimination of PCR amplification. miRs are commonly found in body fluids, such as blood, saliva, spinal fluid and urine. The composition of blood is very complex as compared to other biofluids and hence miR isolation processes cannot be transposed from one fluid type to another. For profiling studies, both plasma and serum contain miR biomarkers [153,154,155,156,157,158]. Plasma is routinely collected in tubes containing anticoagulants, and serum in tubes that promote coagulation. It has been observed that some variables may be introduced during the sample collection process that adds another level of complexity to the current profiling limitations. Other variables may come from the analysis of fasting and non-fasting blood samples and sample storage duration [159,160,161,162]. Recent studies of circulating miRs show conflicting results regarding the relative presence of miRs in plasma compared to serum samples [163,164]. The effect of external variables may be minimized if all sample processing protocols are consistent with sample acquisition, storage and collection within some fixed parameters. Currently, miR extraction methods use guanidium-enhanced phenol:chloroform solution followed by selective solid-phase adsorption of the aqueous phase from the phenol:chloroform mixture onto a mini-column and elution into water or buffer. Several commercial miR isolation kits are available, such as silica based miRVana PARIS kit (Thermo Fisher Scientific Inc., Kalamazoo, MI, USA), miRNeasy Mini kit (Qiagen, Valencia, CA, USA), Qiagen miRNeasy Serum/Plasma kit (Qiagen, Valencia, CA, USA), Sigma-Aldrich marketed Premier microRNA isolation kit (Sigma-Aldrich, St. Louis, MO, USA) and resin based Exiqon MiRCURY RNA isolation kit (Exiqon, Woburn, MA, USA). However, all these isolation kits still employ multiple steps and require access to laboratory equipment for centrifugation, mixing, and freeze-thawing. Further, the extensive sample handling steps may lead to sample degradation by endogenous RNase activity, as well as extremes of pH and temperature [165,166]. Additionally, the presence of low-copy-number miRs makes the isolation process even more challenging, emphasizing the importance of efficient sample processing to minimize loss. Hence, an enclosed integrated unit that can rapidly isolate miR biomarkers from complex samples into a standardized buffer and deliver them to the sensor with minimum human intervention would provide distinct advantages over currently available approaches.

Recent work has focused on development of microfluidic systems capable of analyzing bio-specimens including blood [167,168,169], serum [170], urine [171], nasal fluids [172] and oral rinse [173,174,175]. Researchers have been able to combine the benefits of PCR and microfluidics to develop an integrated system that is able to perform sample loading, cell lysis, amplification and detection on a singular device [173,176]. In another study, an integrated microfluidic continuous flow device was used for detection of biomarkers from oral fluids. In this study, the integrated device was capable of automatically introducing the necessary reagents to perform the detection as well as capable of upstream sample pretreatment [175]. In yet another study, cell lysis, isothermal amplification of target and detection using the naked eye was proposed in an integrated nucleic acid diagnostic device [170]. Researchers have also developed credit card-sized biochips for immune-detection and quantification of a biomarker panel associated with ovarian cancer [177]. Other integrated diagnostic technologies like droplet-based devices [178,179], centrifugal devices [180,181], capillary devices [182,183], paper-based devices [152,184,185], and lateral flow assays [173,176,183,186] have also been developed. In the integrated droplet-based devices, small oil droplets are formed with each droplet acting as a reaction chamber between a target DNA and a fluorescently-labeled molecular beacon. A downstream optical fiber is used to perform detection based on whether or not the droplet fluoresces [178]. Researchers have also developed a portable and fully-automated immunoassay platform into a compact disk technology to test infectious disease from whole blood using centrifugal force [168]. An affinity column and capillary electrophoresis channels were used in an integrated microdevice to detect multiple biomarkers from human blood serum [187]. Such systems used the solid phase extraction principle to capture the target on the microchip column functionalized with antibodies and later elute the target for downstream analysis. While the droplet-based devices, capillary-based and centrifugal integrated systems might offer design and sensitivity advantages, they may be too complex for field applications. On the other hand, paper-based devices and lateral flow assays are designed specifically for field applications and may require minimal infrastructure or additional detection instruments, however they suffer from the major disadvantage of sensitivity. Both of these groups of devices rely on capillary force for the transfer of reagents and target sample and often have a visual readout downstream. However, variability in the sensitivity and selectivity of such devices has been reported in the literature, particularly associated with home-based tests [188]. Commercially-available lateral flow diagnostic devices include GenoType (Hain Lifescience GmbH, Nehren, Germany) and INNO-LiPA (Innogenetics NV, Ghent, Belgium). The GenoType^®^ MTBDR*plus* assay was validated for detection of tuberculosis with high sensitivity [189]. One of the major advantages of the INNO-LiPA lateral flow assay is its ability to detect several targets simultaneously. This assay has been utilized in cervical cancer screening to identify the prevalence and distribution of HPV genotypes in cervical cancer among women in Thailand [190]. In addition to cervical cancer, the INNO-LiPA test was also used to study the distribution of HPV genotypes associated with oral and oropharyngeal squamous cell carcinoma. The capability of the assay to detect several targets was found to be a strength in this study as it was capable of identifying several possible associated genotypes, however, the study found non-consensus in results when the assay was compared with other commercially-available assays [191].

While these microfluidic systems bring the field a needed step closer to cancer screening, several of these platforms involve extensive integration of multiple systems and require peripheral detection systems. The use of complex fabrication systems and numerous assembled modules make it challenging to transition the device to high scale manufacturing. Their complexity also introduces robustness issues due to contamination and target loss that are particularly acute for quantification of low-copy-number miRs. Although some commercially available medical diagnostic products are FDA-approved, like the Focus Dx by Quest Diagnostics (Madison, NJ, USA), Handy Lab by Becton-Dickinson (Franklin Lakes, NJ, USA), Abbott’s i-Stat Corp (Chicago, IL, USA), IQuum by Roche, and Cepheid’s GeneXpert (Sunnyvale, CA, USA), they are still not suitable for low-resource settings [183,192,193,194] due to bulky and expensive instrumentation or high cost per assay. The Cepheid GeneXpert system is probably a true push-button integrated system with sample-to-answer capability; however, the assay for miR screening is currently not commercially available. While the Cepheid GeneXpert system may be adapted for detection of oropharyngeal cancer in developed nations, the high cost of the system makes it unsuitable for detection of cervical cancer which is a significant problem in developing nations.

**Figure 2:**
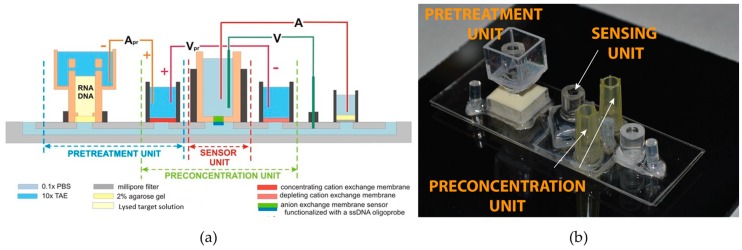
Integrated nucleic acid sensing platform. (**a**) Schematic of an integrated biosensing platform consisting of a pretreatment, preconcentration and sensing units; (**b**) Picture of the integrated biochip. Adapted with permission from [196]. PBS: phosphate-buffered saline; TAE: Tris-acetate-EDTA; ssDNA: single-strand DNA.

In contrast, Slouka et al. and Senapati et al. developed a simple, single-channel integrated diagnostic platform as shown in Figure 2, consisting of a pretreatment unit for extraction of nucleic acid biomarkers, a preconcentration unit to concentrate the extracted nucleic acids (reduces assay time by minimizing diffusion limitation) and a nanomembrane-based biosensing unit for selective detection of target nucleic acids [195,196]. The team utilized the inherent charge of nucleic acid molecules and ion-exchange membranes to develop an integrated multiplexed diagnostics platform capable of processing real biological samples and detecting pathogens in 45 min, often without PCR amplification for overexpressed miR targets [196]. While further validation of this platform using biological and clinical samples is ongoing, the unique features of this system are highlighted below.

The working principle of the pretreatment chip developed by Slouka et al. was based on electrophoretic extraction (by application of direct current voltage) of negatively-charged nucleic acid molecules from the sample reservoir through a thin agarose gel layer into a microfluidic channel as shown in Figure 2a [196]. All neutral, positive, large negative molecules (larger than the pore size of the gel) and debris remain in the sample reservoir.

Downstream from a pretreatment unit was a pre-concentration unit consisting of a cationic exchange membrane. The goal of the pre-concentration unit was to form an ionic plug using electric field and block the passage of the extracted nucleic acids. This resulted in the accumulation of the extracted nucleic acids in a specific location thus improving the sensor sensitivity. The location of the concentrated molecules depended on the flow rate of target molecules in one direction opposed by the ionic plug force in the other direction [196].

**Figure 3:**
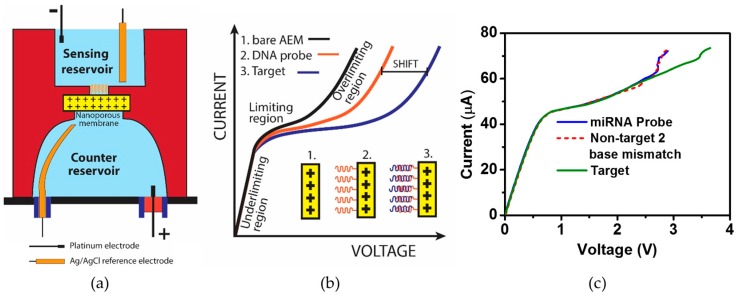
Sensing unit of the integrated platform. (**a**) Schematic showing the ion-exchange nanomembrane sensor; (**b**) Current-voltage characteristics (CVC) showing changes in ohmic, limiting and overlimiting regions for bare anion-exchange membrane (AEM; black), membrane functionalized with oligoprobe (red) and hybridization of DNA/RNA with oligoprobe (blue); (**c**) The specificity of the sensor was challenged by using a non-target sequence differing by only two base pairs compared to the target sequence. Change in CVC was only observed for the target miR sequence indicating the sensor’s capability to distinguish two-base-pair mismatches. Adapted with permission from [195].

As for the biosensing unit for this particular integrated platform, an ion-selective nanomembrane was used for molecular detection of nucleic acid molecules. In prior studies, the group showed the application of anion-exchange membranes for detection of negatively charged molecules using the charge inversion phenomenon [195,196,197,198,199,200]. The sensor works on the principle that molecular probes functionalized to the nanomembrane sensor will hybridize to target nucleic acids and alter current flow through the ion-selective membrane as shown in Figure 3a,b. This property of the ion-exchange membrane has been shown to detect bacteria, viruses and miR associated with oral cancer [195,196,201]. While demonstrating the detection of miR146a associated with oral cancer, the group also demonstrated the sensor’s specificity by showing no change in electrical signal when a non-target miR146b was introduced in the sensing chamber as shown in Figure 3c [195].

**Figure 4:**
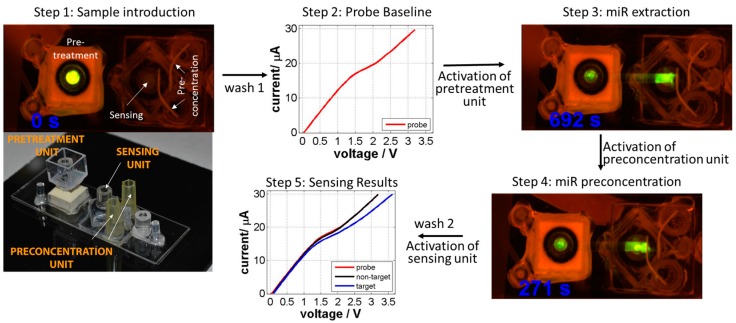
Steps involved in the successful extraction, concentration and detection of miR146a biomarker from oral cancer cell lysate solution. Step 1: The sample is introduced in the pretreatment chamber; Step 2: A baseline CVC is measured for the probe on the sensor; Step 3: An electric field is applied to extract nucleic acid molecules from the pretreatment unit; Step 4: The preconcentration unit is used to concentrate the extracted nucleic acids; Step 5: After allowing target hybridization with the probe, a second CVC is measured to analyze the presence or absence of target.

The integrated platform was constructed by combining these three components using low-cost polymeric polycarbonate materials, thus reducing the overall cost of the assay. The external circuitry eliminates any on-chip micro-electrode fabrication and hence reduces manufacturing challenges and cost. Another advantage of the platform is its modular design which allows for rapid prototyping of the different components. Oral cancer cell line samples were used to successfully verify the platform’s performance for the detection of target miR in under 45 min. Figure 4 illustrates the sequential operation of the sample pretreatment unit to extract nucleic acid biomarker and nanomembrane based pre-concentration/sensing unit to generate a shift in electrical signal upon presence of the target biomarkers. More importantly, repeating the five steps with a non-target resulted in no shift in I-V curve, confirming sensor’s selectivity [196]. This clearly demonstrated the ability of the entire platform to selectively detect short miR molecules from sample introduction to answer in an integrated fashion and can be easily extended for the detection of miR biomarkers associated with cervical and oropharyngeal cancers.

Current research is focused on extension of this microfluidic platform to a panel of miR biomarkers, with insertion of multiple sensors (≈10 targets) into the membrane sensor module and by using a reasonable sample volume. Advantages of this multi-target platform are reduction of miR degradation and loss, removal of inhibitors, and incorporation of a low-cost but sensitive membrane sensor. The key remaining issue is whether the current platform will offer sufficient sensitivity and specificity to quantify low-copy number miRs. If PCR amplification is necessary, a new module must be designed for low-copy number miRs or to quantify under-expressed cohorts. Such modules could be digital PCR or solid-state nanopore sensors, with single molecule resolutions for the lower-copy-number targets. It is hence quite possible that a second microchannel needs to be inserted to divert the sample to a second module. This more complex integration may lead to increased target loss and robust designs must be carefully implemented. The design strategy would again be to minimize the pretreatment and isolation steps, perhaps by dilution to reduce the concentration of the inhibitors. High-throughput digital PCR will allow such dilution and would be an attractive low-copy-number complement to the current high-copy-number microfluidic platform. Nanopore sensors may require preconcentration if the sample is diluted. Extension to multi-targets without PCR amplification would be a significant advance of the current nucleic acid quantification technology and should be the next technical development.

## 6. Future Directions

Significant challenges remain for development of robust biosensors used for disease detection in the developing world. For example, while cervical cancer is consistently decreasing in the U.S., it remains a significant public health challenge in developing nations. There is an unmet medical need for a low cost, easy to use, point-of-care diagnostic that can be used for self-sampling or clinic-based sampling of cervical cells. This would facilitate screen-and-treat in the same clinic visit, providing a major advantage to patients that lack access to transportation. Such a device would also simplify analysis of patient response to surgical procedures such as loop electrosurgical excision procedure (LEEP) and for more routine monitoring of vaccine efficacy.

Any diagnostic platform is only as good as the validity of the biomarkers it detects. Hence, an important future direction is the discovery and approval of more specific miR biomarkers for cervical/oropharyngeal cancer. Redundancy with multiple targets would minimize false positives and negatives. Over-expressed miRs are easier to quantify, provided the specificity is not compromised by other interfering miRs with similar sequences that could compete for the oligo probes. Optimal fragmentation of long HPV DNA will also enhance the sensitivity and selectivity. This will require proper selection of target sequences and fragmentation enzymes. Other than target discovery and selection, more advanced pretreatment units that can remove interfering debris and lipids of certain samples will also advance the platform. Portable instrumentation, large-scale manufacturing designs and field validation would then be the final steps. Like any other new platform, the road to commercial and clinical realization is long and tortuous. However, the unique advantages of emerging nucleic acid biosensor technologies may fundamentally transform health care in low-resource developing countries.

In addition to the examples highlighted, a robust nucleic acid-based biosensor would have utility in a large variety of applications. Development of low cost miR-based screening for recalcitrant cancers with low survival rates such as pancreatic cancer or ovarian cancer may enable population-based screening to improve the incidence of early detection and thereby improve overall survival from these diseases. Portable units would also enable use in environmental applications such as monitoring for invasive aquatic species or for rapid point-of-care detection of emerging pathogens.

## Figures and Tables

**Table 1:** Advantages and disadvantages of traditional cancer screening tools for cervical and oropharyngeal cancers.

Screening Tools	Cancer	Advantages	Disadvantages	Performance
Pap smear	Cervical	Widely used	Expensive Not suitable for low-resource settings Requires trained personnel Long assay time (days)	se: 51% ^a^ sp: 67% ^a^ ppv: 96% ^a^ npv: 8% ^a^
Liquid-based cytology	Cervical	Sample standardization Increased adoption More sensitive than Pap	More expensive than Pap smear Not suitable for low-resource settings Requires trained personnel Long assay time (days)	se: 55% ^a^ sp: 78% ^a^ ppv: 98% ^a^ npv: 10% ^a^
VIA	Cervical	Inexpensive Ideal for low-resource settings Fast results (few minutes)	Requires trained personnel	se: 63% ^b^ sp: 66% ^b^ ppv: 37% ^b^ npv: 85% ^b^
HPV antibody detection	Cervical/Oral	Direct detection of HPV Able to detect genes related to high-risk HPV	Non-specific as not all HPV results in cancer	se: 58% ^c^ sp: 97% ^c^ ppv: 97% ^c^ npv: 58% ^c^
Visual Inspection	Oral	No need of specialized instruments	Dependent on dentist’s experience	se: 93% ^d^ sp: 75% ^d^ ppv: 78% ^d^ npv: 90% ^d^
Tissue biopsy	Cervical/Oral	Highest sensitivity/specificity	Requires trained personnel Long assay time (days) Invasive for patients	Considered gold standard ^e^
Light-based detection systems	Oral	Improved visualization of the neoplastic region Possibility for automated image analysis Instantaneous results	Autofluorescence from non-cancerous cells Not suitable for early screening	se: 53% ^f^ sp: 56% ^f^ ppv: 15% ^f^ npv: 89% ^f^

se: sensitivity; sp: specificity; ppv: positive predictive value; npv: negative predictive value; HPV: human papillomavirus; VIA: visual inspection with acetic acid; ^a^ Performance when compared to tissue biopsy [46]. Note that these are comparison results from a single study; ^b^ Performance when compared to tissue biopsy [47]; ^c^ Performance characteristics for 16L1 peptide-based enzyme-linked immunosorbent assay (ELISA) for serological detection of cervical cancer [48]; ^d^ An older study on performance of unaided visual inspection or oral lesions [49]; ^e^ Performance of other tests were based on the consideration of tissue biopsy as the gold standard; ^f^ Performance characteristics for Vizilite technology when compared to biopsy [50].

**Table 2:** Comparison of nucleic acid-based cancer screening techniques.

Technique	Throughput	Specificity	Sensitivity	Cost/miR [84]	Assay Time
Microarray	High	Moderate	Low	Low	1–2 days
qRT-PCR	Low	High	High	High	Few hours
Next generation sequencing	High	High	High	Low (High/sample)	2–5 days

qRT-PCR: quantitative reverse transcription-PCR; miR: microRNA.

**Table 3:** Comparison of FDA-approved HPV detection assays [120].

Performance Specification	Qiagen^®^ HC2 Assay	Cervista^®^ HPV HR	Cervista^®^ HPV 16/18	Cobas^®^ HPV	Aptima^®^
Detection Target	13 high-risk HPV DNA	14 high-risk HPV DNA	HPV 16 and 18	14 high-risk HPV DNA	E6/E7 mRNA of high risk HPV
Detection Mechanism	Antibody hybridizes to HPV DNA–RNA	Invader Chemistry	Invader Chemistry	PCR + Fluorescence	Transcription-mediated amplification HPV 16/18
Sample Processing	Additional pretreatment needed	Additional pretreatment needed	Additional pretreatment needed	Automated sample extraction	Automated sample extraction
Clinical Sensitivity [112,121,122,123]	≈94%	≈89%	68%–70%	≈93%	>92%
Specificity [112,121,122,123]	>89%	≈91%	62%–80%	≈99%	≈99%
Assay vs. System [117,118,124,125,126,127,128]	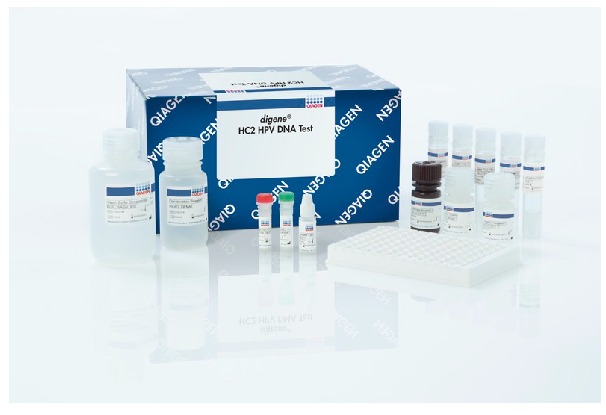 Assay	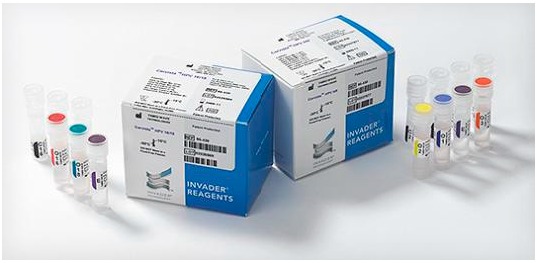 Assay	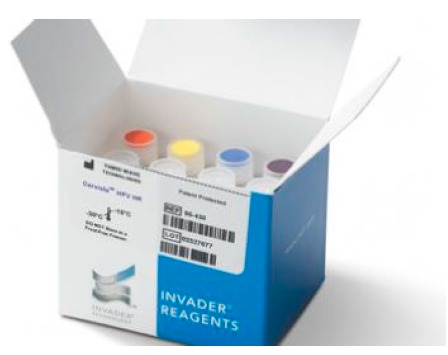 Assay	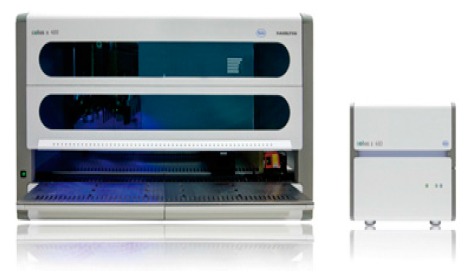 Cobas^®^ HPV System	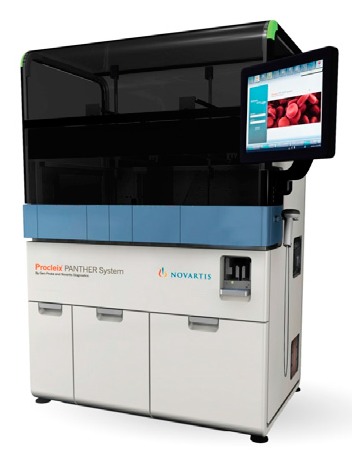 Panther System
Cost [124,125,126,127,128]	$71 per test°	$30 per test°	$30 per test°	$35 per test° + capital equipment	$30 per test° + capital equipment

FDA: Food and Drug Administration; ° Note that the prices mentioned are costs associated with the assays only. Additional labor costs and capital equipment costs may also apply.
